# Using Machine Learning to Compare Provaccine and Antivaccine Discourse Among the Public on Social Media: Algorithm Development Study

**DOI:** 10.2196/23105

**Published:** 2021-06-24

**Authors:** Young Anna Argyris, Kafui Monu, Pang-Ning Tan, Colton Aarts, Fan Jiang, Kaleigh Anne Wiseley

**Affiliations:** 1 Michigan State University East Lansing, MI United States; 2 School of Business University of Northern British Columbia Prince George, BC Canada; 3 Department of Computer Science University of Northern British Columbia Prince George, BC Canada

**Keywords:** antivaccination movement, Twitter messaging, public health informatics, supervised machine learning algorithm, unsupervised machine learning algorithm, qualitative content analysis, data visualization, infodemiology, infodemic, health misinformation, infoveillance, social listening

## Abstract

**Background:**

Despite numerous counteracting efforts, antivaccine content linked to delays and refusals to vaccinate has grown persistently on social media, while only a few provaccine campaigns have succeeded in engaging with or persuading the public to accept immunization. Many prior studies have associated the diversity of topics discussed by antivaccine advocates with the public’s higher engagement with such content. Nonetheless, a comprehensive comparison of discursive topics in pro- and antivaccine content in the engagement-persuasion spectrum remains unexplored.

**Objective:**

We aimed to compare discursive topics chosen by pro- and antivaccine advocates in their attempts to influence the public to accept or reject immunization in the engagement-persuasion spectrum. Our overall objective was pursued through three specific aims as follows: (1) we classified vaccine-related tweets into provaccine, antivaccine, and neutral categories; (2) we extracted and visualized discursive topics from these tweets to explain disparities in engagement between pro- and antivaccine content; and (3) we identified how those topics frame vaccines using Entman’s four framing dimensions.

**Methods:**

We adopted a multimethod approach to analyze discursive topics in the vaccine debate on public social media sites. Our approach combined (1) large-scale balanced data collection from a public social media site (ie, 39,962 tweets from Twitter); (2) the development of a supervised classification algorithm for categorizing tweets into provaccine, antivaccine, and neutral groups; (3) the application of an unsupervised clustering algorithm for identifying prominent topics discussed on both sides; and (4) a multistep qualitative content analysis for identifying the prominent discursive topics and how vaccines are framed in these topics. In so doing, we alleviated methodological challenges that have hindered previous analyses of pro- and antivaccine discursive topics.

**Results:**

Our results indicated that antivaccine topics have greater intertopic distinctiveness (ie, the degree to which discursive topics are distinct from one another) than their provaccine counterparts (*t*_122_=2.30, *P*=.02). In addition, while antivaccine advocates use all four message frames known to make narratives persuasive and influential, provaccine advocates have neglected having a clear problem statement.

**Conclusions:**

Based on our results, we attribute higher engagement among antivaccine advocates to the distinctiveness of the topics they discuss, and we ascribe the influence of the vaccine debate on uptake rates to the comprehensiveness of the message frames. These results show the urgency of developing clear problem statements for provaccine content to counteract the negative impact of antivaccine content on uptake rates.

## Introduction

### Background and Specific Aims

Vaccine-critical social media content has been suggested as a major obstacle to immunizing the public against vaccine-preventable diseases [1-4]. According to a 2019 survey on internet use in the United States, 79% of adults are on social media [5], and a separate health-focused survey conducted in 2019 found that 75% of adults read medical information on social media [6]. Over a 5-year period from 2012 to 2017, the percentage of people seeking medical advice online increased from 8% to 31.5% [7]. Simultaneously, the spread of antivaccine content has accelerated on social media [8], fostering groundless fears about immunization [9].

Exposure to antivaccine content on social media has been associated with delays in and refusal of vaccination [3]. While the development of tailored messages (eg, text messages) has increased immunization rates [10], public provaccine campaigns via social media have yielded limited success, as shown in several systematic reviews of interventions for various vaccines [4,11-19]. Given that there is a methodological barrier to assessing the impact of public campaigns on regional immunization rates, the current literature has not yet fully explicated how the antivaccine movement continues to engage and persuade the public to deny immunization despite provaccine advocates’ counteracting efforts. Therefore, there is a need to compare pro- and antivaccine advocates in terms of the discursive topics they deploy on social media to engage and persuade audiences to accept or deny immunization.

This study comparatively analyzed pro/antivaccine content on social media on the spectrum of engagement persuasion devised based on digital marketing [20-23] and social influence literature [24]. Literature on digital media marketing suggests that the effectiveness of a campaign should be evaluated on a broader spectrum from engagement to persuasion because engaging audiences with content that competes against numerous other sources of content for their attention is a precursor to persuasion [20,21]. Simultaneously, literature on social influence within a large public network (eg, social media) states that public engagement is a foundation upon which one can exert influence on the audience’s attitudes and behaviors [24]. Based on this literature on digital marketing and social influence on a digital network, we define the engagement-persuasion spectrum as *a wide range of discursive strategies devised to influence an audience’s attitudes and behaviors about a target matter on social media*. This spectrum starts with engaging the audience with the content and concludes with persuading the audience to accept the claims included in the content.

As a way of fostering engagement, a greater diversity of discursive topics has been suggested [20]. Antivaccine advocates employ more diverse topics than their provaccine counterparts, and previous researchers have claimed that this results in higher engagement [25-28]. However, the diversity of topics (ie, the number of topics discussed) is not sufficient to harness public engagement [20]. Social media campaigns ought to ensure that the topics discussed are distinct from one another, thus attracting a wider range of individuals with diverse interests, and that discourse surrounding a topic is internally consistent and coherent so they make sense to the public [20]. We herein call the former *intertopic distinctiveness* and the latter *intratopic consistency*.

Persuasion should follow engagement, which in this case is the effort by pro- and antivaccine advocates to encourage the public to accept or deny immunization. Framing vaccines in communications with individuals (eg, parents) has been suggested as a viable option for this purpose [29,30]. However, how the pro- and antivaccine messages fit into Entman’s four dimensions [31] is less known. Entman’s message frames are persuasive techniques used in propaganda in which a speaker tries to predispose the audience to a one-sided view of an issue while downplaying other perspectives. Indeed, antivaccine advocates disproportionately emphasize safety concerns while downplaying the preventive benefits of vaccines. When used consistently, Entman’s frames can induce behavioral and attitudinal changes among audiences [31]. Therefore, identifying how Entman’s four frames are used in pro- and antivaccine content will enable us to gauge how persuasive is each side.

The overall objective of this study was to compare the discursive topics pro- and antivaccine advocates deploy to influence the public to accept or deny immunization on the engagement-persuasion spectrum. Our overall objective was pursued using three specific aims as follows: (1) we classified tweets into provaccine, antivaccine, and neutral categories; (2) we extracted and visualized the intertopic distinctiveness and intratopic consistency of the discursive topics among the pro- and antivaccine tweets classified in (1); and (3) we identified how those topics frame vaccines along Entman’s four framing dimensions. Our rationale for the first specific aim was that an automatic pro- or antivaccine classification is necessary for analyzing discursive topics on each side due to the sheer volume of vaccine-related content created and circulated on social media on a daily basis. Our justification for the second specific aim was that we need an autonomous method that considers the numerous linguistic features included in pro- and antivaccine tweets and extracts topics from both sides without human bias. The achievement of these first two aims explains higher engagement among antivaccine advocates than their provaccine counterparts. Our rationale for the third specific aim was that an analysis of the pro- and antivaccine topics along Entman’s four dimensions would allow us to better understand how antivaccine advocates succeed in persuading the public to reject immunization as compared to their provaccine counterparts.

In achieving these aims, we make several contributions to methodological advances. First, we collected a large coverage of both pro- and antivaccine social media posts that fairly represent both parties [32]. Second, we developed a machine learning (ML)-based automatic classifier of pro- and antivaccine posts and unsupervised clustering for extracting discursive topics. This set of ML algorithms will aid future researchers in assessing the effectiveness of public health campaigns on social media and hence facilitate the successful development of future interventions. Lastly, we conducted a multistep content analysis that combines interpretive (inductive) with objective (deductive) coding to identify the topics within the dimensions of Entman’s four message frames. In so doing, we alleviated methodological challenges that have hindered an analysis of pro- and antivaccine discursive topics from a broader engagement-persuasion perspective.

### Prior Studies on Pro- and Antivaccine Advocates on Social Media

Antivaccine advocates on social media have shown more notable engagement patterns than their provaccine counterparts. On Instagram and Facebook, interaction tends to be higher with antivaccine content than with provaccine content [33,34]. Antivaccine articles are shared more widely than provaccine articles [28]. Although the number of provaccine tweets exceeds the number of antivaccine tweets, the proportion of antivaccine users on Twitter is rising, having nearly doubled from 8.1% to 16% between 2015 and 2018 [35]. Moreover, those who have been exposed to antivaccine content on Twitter and Facebook [34,36] are more likely to disseminate similar antivaccine content. Parents exposed to antivaccine content on Facebook were 1.6 times more likely to consider vaccines to be unsafe [37]. Antivaccine communities are more integrated with users who are undecided about vaccines compared to provaccine users, who remain on the periphery [26].

This higher user engagement has been attributed to a higher diversity of topics included in antivaccine rhetoric compared with provaccine content. Strong themes have emerged among antivaccine communities, and they tend to cover a more expansive and generalizable range of content than provaccine communities [26]. This expansive range of topics is conducive to defining a broad “in group” based on shared values as follows: distrust of the government and pharmaceutical companies, health and safety awareness, the use of natural health and wellness strategies [25-28], emphasis on religion and morality [25,27], and advocacy for individual liberties [25,27]. Antivaccine communities also tend to share news reports and personal narratives among themselves, elevating the visibility and pertinence of select issues across communities on social media [4,28]. Memon et al [38] conducted a network and linguistic analysis of vaccine-related tweets and found that antivaccine communities use more specific, dramatized, and personalized linguistic features, have higher network density, and demonstrate higher echo-chamberness than do provaccine advocates. Furini and Menegoni [39], Faasse et al [40], and Okuhara et al [41] defined the characteristics of topics used by pro- and antivaccine groups such as the tendency for antivaccine sites to focus on vaccine side effects and for provaccine sites to focus on vaccine primary effects.

These seminal works, however, have not yet fully explicated the intertopic distinctiveness or intratopic consistency of discursive topics discussed by pro- and antivaccine advocates, even though these factors are known to foster user engagement [20,42]. In addition, few prior studies have applied Entman’s message framing to explain how antivaccine advocates portray vaccines as harmful rather than beneficial [39-41]. Our suggested comparison between pro- and antivaccine content using the engagement-persuasion spectrum therefore helps explain both why and how antivaccine communities demonstrate higher engagement and affect uptake rates despite opposition from provaccine advocates.

### Hypotheses Development on the Engagement-Persuasion Spectrum

Recent studies on social media marketing posit that a variety of content should be created to actively engage customers in a dialogue with the speaker [20]. This marketing perspective is relevant because pro- and antivaccine advocates compete to keep the audience engaged in their content with the ultimate goal of persuading the public for or against vaccines [26].

Although a diversity of topics in social media content is linked to increased user engagement, simply counting the number of topics discussed is not sufficient [20,42]. Instead, one ought to consider intertopic distinctiveness, which aids in serving a wider array of individuals with various interests [20]. For instance, if provaccine advocates discuss various issues only in the realm of contagious diseases (eg, herd immunity), individuals who believe they have strong immunity (eg, young people) may not engage with such content. The COVID-19 pandemic has made clear the importance of intertopic distinctiveness. Communicating the harms of the viral infection was not enough to encourage some people in their 20s and 30s to comply with the measures of state lockdowns or social distancing in the United States. It is therefore important to develop various distinct topics to attract individuals with different interests (eg, herd immunity, fitness, and lifestyle). Next, intratopic consistency helps the audience make sense of the content, thereby facilitating the achievement of communication goals [20]. Especially in the uncontrolled space of social media, establishing consistency of messaging keeps the audience engaged [20]. Creating a coherent and consistent image of a reference object (in this case, vaccines) by coordinating and connecting messages, arguments, and statements is an integral part of social media communication [20]. Accordingly, we assessed whether antivaccine topics indeed have higher intertopic distinctiveness and intratopic consistency than provaccine topics.

Our next step was to measure the persuasiveness of messages. Prior studies have suggested that message frames are a viable option in terms of counteracting ever decreasing immunization rates. For instance, McGlone et al [29] have studied the possibilities for provaccine framing by health care sources to communicate with parents through text messages. Shoup et al [30] incorporated message framing into social media by observing and categorizing mothers’ conversations on a moderated platform created specifically for patients of Colorado’s health system.

Despite such prior pioneering attempts, these studies have not yet adopted Entman’s four message frames [43-45]. Entman’s message framing refers to the strategic and deliberate selection of content for messages with the purpose of attaching positive or negative meaning to an initially neutral topic [31,44]. Frames can illuminate or downplay specific aspects of an issue so that recipients of the message will begin to view the issue from the speaker’s perspective [31]. Antivaccine advocates emphasize injuries and conspiracies surrounding vaccines so as to convince viewers to consider vaccines unsafe, while provaccine advocates underscore the preventive benefits of vaccines and portray them as public health assets [46]. Entman [31] asserts that speakers frame an issue through (1) defining it, (2) interpreting its cause, (3) morally evaluating it, and (4) recommending a remedy to it. Parties that consistently use these four message frames have a greater influence on the majority of receiving audiences [31], and this influence induces attitudinal and behavioral changes [24]. Therefore, we employed Entman’s four message frames to analyze whether and how antivaccine advocates employ these four frames more comprehensively than provaccine advocates.

## Methods

### Overview

We adopted a multimethod approach to analyze discursive topics in large-scale vaccine debates on public social media sites. Our approach combined (1) large-scale balanced data collection from a public social media site (ie, Twitter), (2) the development of a supervised classification algorithm for categorizing tweets, (3) the application of an unsupervised clustering algorithm for identifying prominent topics discussed on both sides, and (4) multistep qualitative content analysis for identifying the prominent discursive topics and how vaccines are framed in these topics.

### Step 1: Data Collection

Before and throughout our data collection, we identified, refined, and verified the keywords used to reach a large coverage of pro- and antivaccine tweets during our data collection period. Prior to embarking on the data collection, we studied previous academic and popular literature to identify relevant keywords and performed weekly tests by retrieving tweets using the search terms to ensure that they remained relevant. In particular, from previous academic literature [25] and popular press articles about the vaccine debate (from the *Washington Post*, the *New York Time*s, and *Time* magazine from January 1, 2016, to September 1, 2019), we initially identified a list of 81 keywords related to the vaccine debate on Twitter.

Using these keywords, we then collected data every day in October 2019 and checked to determine how many tweets were retrieved on a weekly basis per keyword. From these weekly analyses, we eliminated 29 keywords for which the median weekly count of tweets retrieved was zero, because the absence of tweets retrieved by these keywords for an entire week indicated that these keywords were no longer relevant. Finally, using the remaining 52 keywords, we collected tweets every day in November 2019.

During our data collection in November 2019, we investigated whether any new topics or trending hashtags related to vaccines that had not been included in our set of keywords had emerged. To do so, we referred to the list of the top 50 trending topics on Twitter, which has been used by prior researchers (eg, Zubiaga et al [47]) to identify popular topics that trigger wider conversations on Twitter. Following Zubiaga et al [47], we checked the top 50 trending topics and hashtags for each day in November 2019, but no new vaccine-related topics emerged. Because Twitter was the source of our data collection, the absence of emerging vaccine-related hashtags in the Twitter top 50 trending topics during our data collection period suggests that our data collection is comprehensive, up to date, and relevant. A list of the 52 keywords is provided in Multimedia Appendix 1.

Our use of 52 keywords could have led to repeated collection of the same tweets if we had not carefully tracked and eliminated them. For instance, two of our keywords, *vaccine* and *sb276*, could retrieve a tweet such as “End Vaccine Tyranny now! End SB276” twice. To avoid redundancy in our data collection, we gathered the unique tweet IDs (tweet_ids) for each post. We kept track of all tweet_ids that we encountered each time we retrieved tweets using a keyword. In the case of collecting a retweet, which also contained the original tweet, we checked the original tweet to see if its tweet_id matched one that we had already collected. If it did not, we added the unique tweet_id to our list and saved the text of the tweet to a file. If the tweet_id was in our list, it was determined to be redundant and was not collected. The total number of tweets collected was 39,962 (11,103 provaccine, 8169 antivaccine, and 20,691 neutral tweets)

### Step 2: Automatic Tweet Classification Algorithm

Next, we annotated tweets to construct our training set. Our initial annotation involved two members of the research team working simultaneously to ensure the correctness of annotations. The two coauthors communicated throughout the annotation process to resolve any disagreements and ambiguities in the annotations and to prevent any errors.

To improve generalizability and alleviate the researchers’ bias in our annotations, we also employed an independent annotator who was not aware of the study’s hypotheses to label a sample of our tweets. This independent coder was thoroughly trained by a member of the author team. Upon completion of the training, the coder was given 300 tweets to label as pro- or antivaccine. The set was an equal split between 150 pro- and 150 antivaccine tweets that had previously been annotated by the authors. We chose 300 tweets based on Durivage’s convention for adequate sample sizes to validate the annotations [48] within a 5%-10% margin of error, assuming a 95% CI. The interrater reliability, measured with the Cohen kappa agreement statistic [49], was 0.83, indicating the highest range of Cohen kappa agreement between the two sets of labels. Our final annotated data set contained 5611 labeled tweets, consisting of the following classes: antivaccine (n=1550), provaccine (n=1639), and neutral (n=2422). The “neutral” class refers to the set of tweets that were neutral to vaccines or unrelated to vaccines even though we had collected tweets using vaccine-related keywords as described in *Step 1: Data Collection*.

The annotated tweets were then used to train a classifier for labeling the vaccination stance of the tweets. For reproducibility purposes, the Jupyter notebook containing our Python code and the results of its execution run can be obtained online [50,51]. First, each of these annotated tweets was preprocessed to generate its feature vector representation. Specifically, we applied NLTK’s TweetTokenizer function to segment each tweet into a set of individual tokens (eg, terms, hashtags, and mentions). Terms corresponding to stop words were automatically removed using NLTK’s stop word list augmented with our own list of stop words [51]. A feature vector was then constructed for each tweet by applying the CountVectorizer function from Python’s Scikit-Learn library. This function takes as input a tweet message and returns a vector of frequencies for each unigram, bigram, hashtag, or mention that appears in the tweet. After preprocessing, each tweet was represented by a feature vector of length 15,948.

Once the feature vector for each tweet was constructed, we used Scikit-Learn’s KFold split function to partition the data into 10 disjoint folds so that we could apply 10-fold cross-validation to train and evaluate our classifier. To do so, we iteratively chose nine of the 10 folds to be our training set while leaving the remaining fold out as test data. Using Scikit-Learn’s l1-regularized logistic regression classifier (with its default hyperparameter value as a regularizer), we trained a model on the training set and applied it to the withheld test data. This process was repeated until each fold was used exactly once as the test data. As the class distribution was potentially imbalanced, we also applied the oversampling technique on the training set to ensure that the induced model was not biased toward accurately predicting the larger class only. This was accomplished by resampling the training examples from the smaller classes (ie, pro- and antivaccine) until every class had an equal proportion in the training data. The logistic regression classifier was then trained on the balanced training data, and its induced model was then applied to the withheld test fold.

Logistic regression is a binary classifier for estimating the conditional probability that an input feature *x* belongs to class *y* using the following equation:

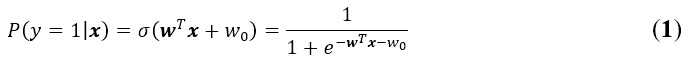



where *σ*(*z*) is known as the logistic function, and {***w***, *w_0_*} are the model coefficients. The coefficients were estimated during training using the maximum likelihood estimation approach. This approach can be described as follows. Let {(***x***1, *y*1), (***x***2, *y*2), …, (***x***_N_,*y*_N_)} denote the training set of *N* labeled tweets. The logistic regression classifier was trained to minimize the following l1-regularized negative log-likelihood function:

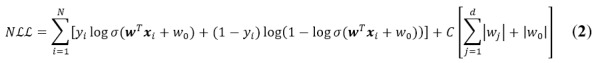



The l1-regularization penalty was used to prevent the model from overfitting the training data. We applied the default hyperparameter value (*C*=1) from Scikit-Learn’s implementation of logistic regression as our regularization penalty. Although it is possible to obtain better results with more careful hyperparameter tuning, the default option was found to be sufficient to produce high accuracy. This is because the number of training examples was sufficiently large to ensure that the test accuracy was quite stable. For example, the test accuracy values varied only slightly between 87% and 91%, as λ varied by two orders of magnitude from 0.1 to 50.

Furthermore, because there were three types of classification labels (provaccine, antivaccine, and neutral), the classifier used the strategy of one versus all to train three binary models to predict each class. Specifically, each binary model was trained to distinguish the tweets of one label (eg, provaccine) from the other two categories. In the prediction step, the classifier applied all three models to each given tweet and assigned it to the class label with the highest aggregated confidence score.

We evaluated the performance of the logistic regression classifier using stratified 10-fold cross-validation. The classifier showed high overall classification accuracy of around 90.1%, which is the percentage of all labeled tweets predicted correctly by the classification models. The detailed classification results for the three categories are shown in the confusion matrix in Table 1.

In addition, we report the precision, recall, and F-measure of the classifier for each tweet class in Table 2.

The results shown in Table 1 can also be aggregated to analyze the classifier’s performance in terms of distinguishing between tweets that are either pro- or antivaccine and those that belong to the neutral class. The confusion matrix for the two categories is given in Table 3, with an accuracy of around 96.2%.

The precision, recall, and F-measure of the two categories are shown in Table 4.

**Table 1:** Confusion matrix for the three classes of tweets.

Actual class	Predicted class		
	Antivaccine	Provaccine	Neutral
Antivaccine	1344	166	40
Provaccine	175	1364	100
Neutral	25	48	2349

**Table 2:** Precision, recall, and F-measure for the three classes of tweets.

Class	Precision	Recall	F-measure
Antivaccine	87.0%	86.7%	86.9%
Provaccine	86.4%	83.2%	84.8%
Neutral	94.4%	97.0%	95.7%

**Table 3:** Confusion matrix for the binary classification of tweets.

Actual class	Predicted class	
	Provaccine or antivaccine	Neutral
Provaccine or antivaccine	3049	140
Neutral	73	2349

**Table 4:** Precision, recall, and F-measure for the binary classification.

Class	Precision	Recall	F-measure
Provaccine or antivaccine	97.7%	95.6%	96.6%
Neutral	94.4%	97.0%	95.7%

Note that if we had explicitly trained a logistic regression classifier to distinguish between the two categories (provaccine or antivaccine vs neutral) instead of simply aggregating the results from Table 1, we would have obtained a similar test accuracy of around 96.1%.

Finally, we retrained the l1-regularized logistic regression classifier on the entire 5611 labeled tweets and applied them to the Twitter data we collected for November 2019. The final distribution of the classified tweets was as follows: provaccine, 11,103; antivaccine, 8168; and neutral, 20,691.

### Step 3: Topic Analysis Using K-Means Clustering

#### Identification of Discursive Topics on Each Side of the Vaccine Debate

Next, we extracted the topical clusters of the pro- and antivaccine tweets that had been downloaded and classified as described above. Specifically, we used the K-means algorithm in the Scikit-Learn Python package [52]. We chose K-means clustering because unlike other algorithms, it has high stability when employing a large amount of data with many dimensions [53]. Clusters derived from the K-means clustering algorithm contain common words mentioned at a similar frequency rate. Thus, each cluster shows a group of words that appear together frequently, comprising a topic of emerging tweets.

To determine the number of clusters (*k*) in both data sets, we measured their silhouette coefficients. The silhouette coefficient is a measure of cluster cohesion that considers the within-group and between-group distances between the members of each cluster. If the silhouette coefficient is 1, then the members in the same cluster are closer to each other than to those belonging to other clusters. If it is −1, then the components of the clusters are completely misclassified. If it is 0, then the clusters are not well separated because their within-group and between-group distances are close to each other [54].

**Figure 1:**
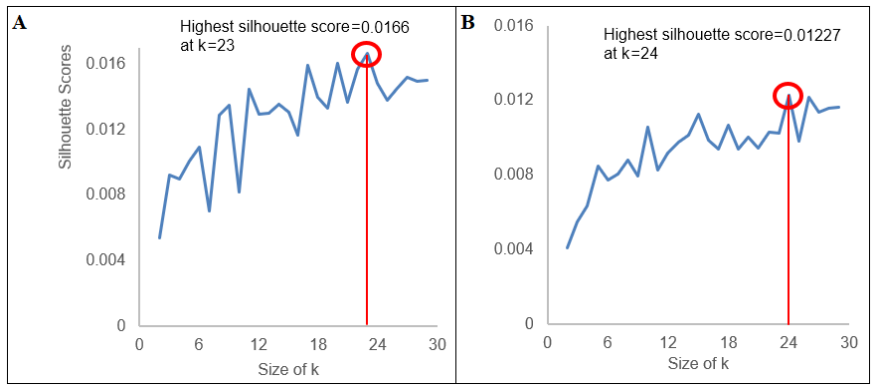
Determining the optimal cluster size (k) for (A) provaccine and (B) antivaccine tweets.

In Figure 1A and B, we plotted the average silhouette coefficients for *k*=0 to *k*=30 in our data. For provaccine tweets, the silhouette score was the highest at *k*=23 (silhouette=0.0166). Thus, we used *k*=23 as the number of clusters (topics) for provaccine tweets. Among antivaccine tweets, *k*=24 had the highest silhouette score (0.0123).

**Figure 2:**
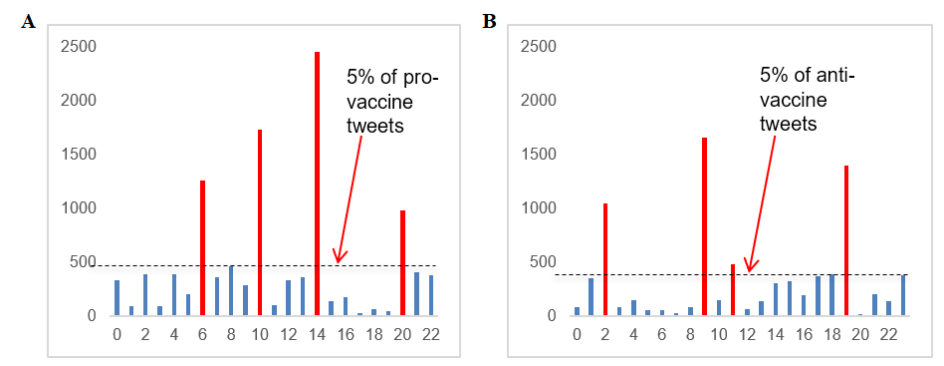
Provaccine (A) and antivaccine (B) cluster counts. In A, clusters 6, 10, 14, and 20 each hold over 5% of the tweets in the data set. In B, clusters 2, 9, 11, and 19 each hold over 5% of the tweets in the data set.

Using these *k* values for the analysis, we identified the prominent clusters of both pro- and antivaccine tweets as those having at least 5% of the total tweets in the data set. For the provaccine tweets, this included clusters with a count higher than 472. For the antivaccine tweets, this included clusters with a count higher than 396. There were four prominent provaccine clusters and four prominent antivaccine clusters. Figure 2 shows the counts for each of these prominent clusters.

Prior to proceeding to the subsequent analyses, we noticed that the top 50 words in three clusters on each side consisted entirely of Twitter handles. These were provaccine clusters 3, 16, and 18 and antivaccine clusters 3, 5, and 7. This suggests that these clusters entail closed-loop tweets and responses to those tweets rather than open dialogues among the public. Consider a long thread of tweet exchanges between two people, @JohnDoe and @JaneDoe, as an example. These clusters represent closed-loop conversations between individual accounts rather than discussion topics among a large group of vaccine advocates, and hence, they are not included in the subsequent analyses.

#### Intertopic Distinctiveness and Intratopic Consistency

Among the selected clusters, we examined the intertopic distinctiveness and intratopic consistency. The former is depicted as the distance between circles, and the latter is depicted in terms of the radius of each circle. The distance between clusters refers to the intertopic distinctiveness. A larger distance indicates distinct topics in the connected pair of clusters, while a smaller distance represents indistinct topics. The distance was calculated as follows:

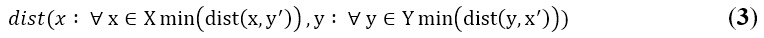



where *x* and *y* refer to data points (tweets) in the clusters X and Y, and *x′* and *y′* refer to the centers of clusters X and Y. A distance score of less than 1 means there is significant overlap between the connected pair of clusters, and a score of 1 or greater indicates that the clusters are distinct.

The radii of the circles of clusters measure the intratopic consistency. A larger circle is associated with more inconsistent tweets in the cluster, and a smaller circle is associated with more consistent tweets in the cluster. The radius of each circle was calculated as follows:

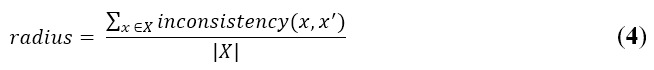



where *x* refers to tweets as data points in the cluster X. The inconsistency (*x*, *x′*) is the separation between the data points *x* and *x′*, where *x′* is the center of the current cluster X. |X| is the total number of elements in cluster X.

### Step 4: Qualitative Content Analysis

In addition to calculating intertopic distinctiveness and intratopic consistency, we applied a two-phased qualitative content analysis to the predominant clusters to identify the message frames used by the pro- or antivaccine clusters. In Figure 2, we selected four predominant provaccine clusters and four predominant antivaccine clusters, each of which have over 5% of the total collected tweets. Our two-phased content analysis consists of first identifying the main topics that appear in each of the predominant clusters and then identifying the frames used in each of the identified topics. This multistep coding was conducted because Entman [31] asserted that message frames should be identified from the topics, not from individual messages such as tweets. In the first phase, we extracted the main discursive topics for each prominent cluster, and in the second phase, we identified the framing used for each topic. Specifically, in the first phase, we exploited the advantages of inductive coding, whereby new concepts emerging in the clusters were identified [55]. In the second phase, we developed a detailed coding scheme to match our clusters to Entman’s (1993) framework [55,56]. Our coding scheme included explicit definitions, examples, and procedures for each category, noting “exactly under what circumstances a passage can be coded with a category” [57]. The coding scheme is presented in Multimedia Appendix 2. Additionally, we employed a second independent coder who had no knowledge of the hypotheses. The interrater reliability with Cohen kappa agreement statistic was 0.83, indicating the highest range of Cohen kappa agreement between the coder and the authors’ categorization [49].

## Results

### Results From K-Means Clustering: Visualization and Significance Testing of Intertopic Distinctiveness and Intratopic Consistency

**Figure 3:**
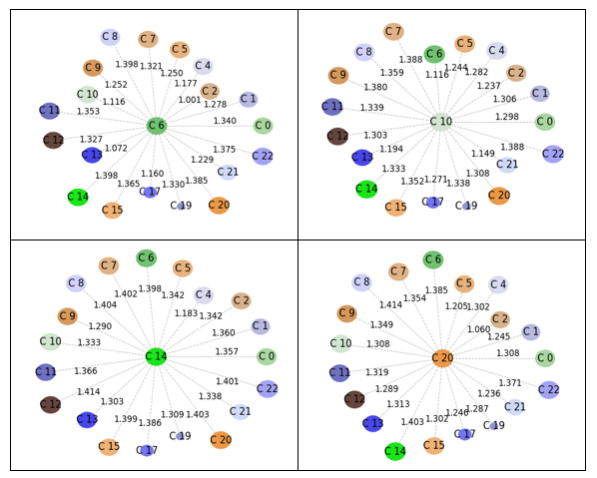
Distance between prominent provaccine clusters and the rest of the clusters.

**Figure 4:**
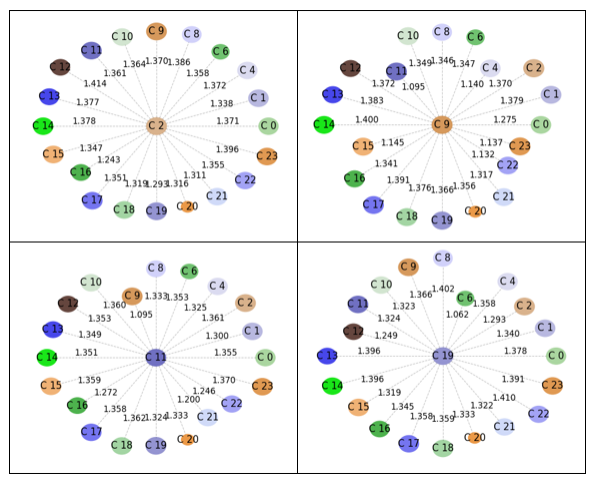
Distance between prominent antivaccine clusters and the rest of the clusters.

From Figure 1, we took 20 provaccine clusters and 21 antivaccine clusters, excluding three on each side that were comprised of Twitter handles only, as described in the previous section *Identification of Discursive Topics on Each Side of the Vaccine Debate*. Additionally, as noted earlier in the same section, we took four prominent clusters (clusters with over 5% of the total tweets) from each of the pro- and antivaccine sides as shown in Figure 2. Then, we plotted the prominent clusters in relation to the rest of the clusters within the respective side of the vaccine debate to visualize intertopic distinctiveness and intratopic consistency, as shown in Figures 3 and 4. Specifically, we positioned the prominent clusters in the center of each chart and related them to the rest of the clusters on their respective sides.

Figures 3 and 4 show that the provaccine clusters were less distinct among themselves than were the antivaccine clusters. We hereafter label the provaccine clusters as Pro-C[cluster number] and the antivaccine clusters as Anti-C[cluster number]. The shortest distance was found between Pro-C6 and Pro-C2, and the distance score was 1.001. The shortest distance found on the antivaccine side was 1.095 between Anti-C11 and Anti-C9. In addition, the mean distance between the prominent provaccine clusters (clusters 6, 10, 14, and 20) and the rest of the clusters was 1.30 (SD 0.09). The mean distance between the prominent antivaccine clusters (clusters 2, 9, 11, and 19) and the rest of the clusters was 1.33 (SD 0.07). Our *t* test result showed that there was a significant difference in the distances between the pro- and antivaccine clusters (*t*_122_=2.30, *P*=.02 [two-tailed]).

Next, we compared the radii difference between the pro- and antivaccine clusters as a measure of intratopic consistency. As noted above, a smaller radius is associated with more consistent tweets within a cluster. The provaccine cluster radius mean was 0.91 (SD 0.14), and the antivaccine cluster radius mean was 0.95 (SD 0.08). We conducted a one-tailed *t* test given a prior study suggesting the higher density and echo-chamberness of antivaccine advocates [38]. There was no significant difference between the radii of the provaccine and antivaccine clusters at the significance level of .05 (*t*_40_=0.99, *P*=.33 [two-tailed]).

### Results From the Qualitative Content Analysis: Message Frames Used by Pro/Antivaccine Advocates

Tables 5 and 6 show the results of our two-phased content analysis. Our inductive coding results revealed two findings that had received less attention in the literature. First, provaccine advocates engaged in attacking antivaccine advocates (Pro-C14), and this topic had the most tweets (2450/11,103, 22.1%; over one-fifth of provaccine tweets). It is well known that antivaccine advocates attack government policies and pharmaceutical companies for being profit driven; however, it is less known that provaccine advocates also engage in condemning antivaccine advocates, except for the following two studies. A thematic analysis of the Australian provaccine movement identified hostility among provaccine advocates toward those who do not share their views [58]; however, this study was limited to the Australian context only. A qualitative interview with antivaccine mothers and women showed that antivaccine advocates believed that they were being stigmatized [9], but this study did not show how provaccine advocates’ hostility was manifested in tweets. Our result showed not only that provaccine advocates criticize antivaccine advocates, but also that this criticism is the most prominent message conveyed by provaccine advocates on Twitter. This result provides a significant implication. Condemning antivaccine advocates can backfire and can only aggravate their mistrust of medical professionals and make their movement resilient [59]. Second, antivaccine advocates suggest a larger conspiracy theory beyond the connections between pharmaceutical industries and the government, including insurance policies, prescription drugs, and opioids (Anti-C9).

**Table 5:** Message frames used in prominent provaccine clusters.

Cluster	Count	Examples	Phase 1 inductive coding: Common topics found in each cluster	Phase 2 deductive coding: Entman’s four message frames
Pro-C6	1256 tweets	*At least 115 countries have HPV vaccine in their normal routine vaccination. The vaccine prevents against HPV which causes cervical cancer. #SABCNews*	Vaccine efficacy (preventive benefits)	(4) Suggest efficacy of vaccines as remedies
Pro-C10	1732 tweets	*Reasons why I get my flu shot: -not a fan of the flu -my mom is immunocompromised from cancer -some people are allergic and can’t get the vaccine -other people are immunocompromised -I trust science and scientists.*	Vaccine saves the vulnerable and the immunocompromised	(3) Moral judgment of the vaccines as creating herd immunity (social good)
Pro-C14	2450 tweets	*Dear antivaxxers… I’m busting my ass in grad school working on fungal vaccine development bc I wanted a creative way to poison the masses? I woulda just started a cult if that was my goal... xo A pissed off scientist.*	Criticizing antivaccine advocates	(2) Identify antivaccine advocates as the ones who cause the problem
Pro-C20	984 tweets	*Hey pls get vaccinated because i know at least three people at my school who aren’t vaccinated simply because they don’t want to and not because of any legitimate reason.*	Encouraging vaccine mandates for school children	(4) Suggest mandated vaccines as remedies

**Table 6:** Message frames used in prominent antivaccine clusters.

Cluster	Count	Examples	Phase 1 inductive coding: Common topics found in each cluster	Phase 2 deductive coding: Entman’s four message frames
Anti-C2	1044 tweets	*If education is the ticket to success #vaccines are the perfect tool to widen the class gap. While vaccine-free rich kids make the most of learning opportunities vaccines doom others to peonage by lowering their IQ and making it tough to function let alone excel in school.*	Advocating exemption for mandatory school immunization	(4) Suggest vaccine exemptions as remedies
Anti-C9	1654 tweets	*The CDC FDA and NIH excel at their core mission of spreading chronic ailments through tainted vaccines to generate huge profits for Big Pharma and Big Medicine.* *@DemocratFed @FloBo2018 Big Pharma is immune from lawsuit spends more $$$ on lobbyists than any other industry and the CDC has been caught covering up data from studies. Only mind controlled slaves would support vaccine mandates. Are you a slave?*	Corrupted connection between pharmaceutical companies and the government (especially Democrats) Overarching conspiracy theory connecting prescription drugs, insurance, and opioids	(3) Moral judgment about the health care system as profit driven
Anti-C11	479 tweets	*So Pharma is in a rush to wipe out the control group to reach 100% vaccination rates before people wake up. Anyone who supports these mandatory vaccine bills (A2371A in NY SB 276 in CA etc.) is engaged in racketeering crimes against humanity. There will be trials.*	Schemes of pharmaceutical companies and injuries to children	(2) Identify pharmaceutical companies as causing the problems
Anti-C19	1397 tweets	*The #vaccine is safe Doc insists the virus is contagious and dangerous. Mom says she must check. She later learns the vaccine carries the risk of twisting the bowel requiring surgery. The momentary rush of pleasing a doctor isn't worth a lifetime of suffering for your baby.*	Vaccine injuries and safety concerns	(1) Define the problems as unsafe vaccines that cause injuries

Our deductive coding results indicated that provaccine themes can be classified into three of Entman’s message frames, but one category, “defining the problem based on a cultural value,” was missing. In other words, provaccine advocates do not clearly define the current problem, thereby failing to communicate the urgency of the matter to the public. Instead, they identify antivaccine advocates as the cause of the problem (Entman’s frame #2, Pro-C14) and make moral judgments, for example, that vaccines are needed to create herd immunity (Entman’s frame #3, Pro-C5). They also suggest vaccine efficacy and school mandates as remedies to the problem (Entman’s frame #4, Pro-C6 and Pro-C20).

In contrast, antivaccine advocates clearly define the current problem, namely the connection between pharmaceutical companies and policy makers (especially Democrats) (Entman’s frame #1, Anti-C9). They also identify pharmaceutical companies as causing the problem (Entman’s frame #2, Anti-C11), seek to increase exemptions to mandated vaccines for public school entry (Entman’s frame #3, Anti-C2), and make moral judgments about vaccination policies as causing injuries and endangering children’s safety (Entman’s frame #4, Anti-C19).

These findings suggest that provaccine advocates do not use message frames as comprehensively as antivaccine advocates do in terms of Entman’s four frames. In particular, while provaccine advocates identify the cause of the problem, make moral judgments, and suggest remedies for the problem, they do not clearly state what this problem is. In contrast, antivaccine advocates provide a compelling statement of the current problem (vaccine injuries) in addition to using Entman’s three other frames.

## Discussion

### Summary of the Findings

In this study, we aimed to comparatively analyze discursive topics in pro- and antivaccine content on the engagement-persuasion spectrum. Our overall objective was pursued with three specific aims as follows: (1) the development of an ML algorithm for automatic classification of pro- and antivaccine tweets, (2) the proposal of an unsupervised ML algorithm for topic analysis (ie, intertopic distinctiveness and intratopic consistency), and (3) the identification of frames used in these topics along Entman’s four dimensions. Our results indicated that antivaccine advocates have significantly higher intertopic distinctiveness than provaccine advocates, but there was no difference between the two groups in terms of intratopic consistency. Our results also indicated that antivaccine advocate messages employ all four major frames that are known to be persuasive, while provaccine advocate messages fail to define the problem.

The first result on the higher intertopic distinctiveness explains the higher engagement among antivaccine communities on social media than that of provaccine advocates, as reported in the current literature [26]. Audiences’ higher engagement in a topic is the first step to inducing behavioral changes favorable to the topic [60]. The higher intertopic distinctiveness of the antivaccine advocates’ topics helps explain how engaging the antivaccine content is, keeping the antivaccine movement resilient and even thriving despite numerous efforts to counteract their messages. The first finding and its implications therefore help us fulfill our first specific aim. The second result suggested a reason why large-scale public provaccine campaigns on social media have rarely been associated with increasing vaccine uptake. Provaccine advocates do not clearly define the current problem; instead, they focus on criticizing and morally judging antivaccine advocates, as well as suggesting remedies. The absence of a clear problem statement limits their capacity to communicate the urgency of the matter at hand. This second result thus fulfills our second specific aim. We must note that no significant difference in intratopic consistency was discerned between pro- and antivaccine content, in contrast to our expectation. We attribute this nonsignificant result to the use of dramatized linguistic features [38] and the frequent references to personal anecdotes and news articles by antivaccine advocates [4,28]. Such varied linguistic features and stories employed by antivaccine advocates could explain why we did not find their topics more consistent than those of their provaccine counterparts. We discuss this point further in the subsequent section *Limitations of the Study and Suggestions for Future Research*.

### Contributions to Knowledge Advancement and Methodology Development

This study contributes to both methodology development and knowledge advancement. First, we developed an ML algorithm that automatically classifies tweets into three classes as follows: provaccine, antivaccine, and neutral. This algorithm has a high accuracy rate (over 90%), which is among the highest for existing algorithms developed for vaccine debates on social media [61,62]. Further, unlike previous work that adopted a one- or two-category classification [34,46], our inclusion of the third category (ie, neutral) screened out irrelevant and neutral tweets with an accuracy rate of 96.2%, allowing us to focus on pro- and antivaccine content only. This algorithm will benefit future researchers who wish to build a public database for social media vaccine debates.

Second, we proposed a way to operationalize and visualize the topics of vaccine debates using K-means clustering. Specifically, our visualization methods can be used to depict intertopic distinctiveness (ie, the distinctiveness of each topic in relation to other topics) and intratopic consistency (ie, the consistency of the themes discussed in each topic). Although the wide variety of topics among antivaccine advocate communities has been noted in earlier studies [25-28], their intertopic distinctiveness and intratopic consistency have not been noted. This study is the first to show that antivaccine topics are distinct from one another, which potentially makes the antivaccine content more engaging to a wider range of individuals with idiosyncratic interests. Future researchers and public health officials may employ these new visualization tools in their efforts to assess the effectiveness of any large-scale health communication campaign.

Third, we devised a two-phased qualitative content analysis whereby we first extracted the prominent topics of each cluster and then identified the message frame employed in that topic following the widely accepted procedure for qualitative content analysis [57]. We also developed a detailed coding scheme and employed an independent coder to ensure the reliability and objectivity of our coding [57]. These coding sheets can benefit future researchers who aim to analyze the topics of the vaccine debate in-depth and develop interventions for disseminating provaccine messages [26].

These methodological advances enabled us to contribute to knowledge advancement in the social media vaccine debate. Many prior studies have examined different patterns of engagement and the diversity of topics between pro- and antivaccine advocates on social media [26,38-41,63-65]. However, comparisons from the broader engagement-persuasion spectrum remain unexplored. In particular, the intertopic distinctiveness and intratopic consistency among the two sides have not yet been compared, even though they are measures of social media users’ engagement with a topic. In addition, Entman’s message framing has not been applied to vaccine debates on social media, although message framing is one of the fastest growing topics in interpersonal vaccine communication [46]. The integrated analyses in this study help identify reasons for the findings reported in prior studies, specifically why antivaccine communities demonstrate higher engagement [39-41,63,64], density, and echo-chamberness [26,38] and how antivaccine advocates successfully dissuade the public from immunization despite opposition from their provaccine counterparts.

### Limitations of the Study and Suggestions for Future Research

The advantages of our study come with several limitations. First, we collected tweets only in November 2019, during which survivorship bias and semantic shifts could have occurred in the vaccine debate on Twitter. To mitigate potential issues with survivorship bias, we selected November, which is during the peak season for antivaccine posts on Twitter [62], to properly represent the antivaccine movement on Twitter. To mitigate likely issues with semantic shifts, we comprehensively chose keywords that were relevant to the vaccine debate prior to embarking upon the data collection and verified that we covered the most current keywords throughout the data collection period.

Our data collection from only Twitter is the second limitation. However, Twitter is one of the most commonly adopted data collection sites for vaccine debates due to its advantage as a public microblogging site where anyone in the public can join in the vaccine debate [62]. Other social media sites, such as Facebook, can be an option but have stricter privacy settings that prohibit researchers from downloading users’ posts. However, we acknowledge that an analysis of more diverse social media may reveal differences unique to each platform.

Third, we analyzed only textual tweets, although social media vaccine debates often employ visual components in their posts [66]. As more social media become visual rather than textual, it will become important to understand how these images deliver a message, and it may be that these topics are different from the ones conveyed in text [66]. These limitations can be overcome by future researchers who expand their data collection for a longer period of time from multiple social media platforms and who develop multimodal algorithms to analyze both text and images.

Fourth, we made an assumption that pro/antivaccine advocates attempt to persuade the public to accept or deny immunization, not each other. As the intended audience for tweets is unclear on social media, this assumption is a potential limitation of this study. However, prior studies have shown that pro/antivaccine advocates are less likely to try to persuade each other due to confirmation and selection biases [26,34]. Rather, pro/antivaccine advocates are more likely to persuade undecided individuals in the general public given the larger presence of undecided individuals (74 million out of 100 million Facebook users) compared to pro/antivaccine advocates on a social media platform [26] and given the main purpose of using Twitter being reaching and persuading a larger audience [67].

Finally, our *t* test result did not show a significant difference between pro- and antivaccine content in terms of intratopic consistency. We attribute this nonsignificant result to antivaccine advocates’ use of various linguistic features [38] and frequent references to secondary sources [28], which may have interfered with our assessment of the intratopic consistency of antivaccine content. One explanation for such nonsignificant results would be that the varied messages seen in antivaccine content make the audience allocate more cognitive capacity to make sense of messages than they would with identical messages, thus making the content more engaging [42]. The current literature on social media marketing has not yet reconciled the conflicting findings between the effectiveness of consistent messages and varied messages for engagement. A comparison between the two may be an opportunity for future researchers.

### Conclusion

Although digital networks have brought several important benefits to public health [10], they have facilitated the propagation of vaccine misinformation. We proposed ML algorithms for automatically classifying a large number of vaccine-related tweets into three classes (provaccine, antivaccine, and neutral), used K-means clustering to quantify and visualize the characteristics of each side of the vaccine debate, and used a two-phased qualitative content analysis to compare both sides of vaccine activism from an integrated communication perspective. Our results indicate that antivaccine content has higher intertopic distinctiveness and frames vaccines along Entman’s four dimensions. These results provide an explanation for the higher engagement among antivaccine advocates and emphasize the urgency of developing a clear problem statement for provaccine content to counteract decreasing immunization rates.

## Appendix

Multimedia Appendix 1 Keywords used for downloading tweets.

Multimedia Appendix 2 Qualitative coding scheme.

## Abbreviations

ML: machine learning
