# Supplementary material for: Using Machine Learning to Compare Provaccine and Antivaccine Discourse Among the Public on Social Media: Algorithm Development Study
Source: JMIR Public Health Surveill. 2021 Jun 24;7(6):e23105. doi: 10.2196/23105 (PMC8277307; doi:10.2196/23105)
Supplement: Multimedia Appendix 1 [file publichealth_v7i6e23105_app1.docx]

**Multimedia Appendix 1.** Keywords used for downloading tweets.

| **Original list**  **Total number:** 81  **Removed keywords**  **Number:** 29 | Vaccine, vaccinated, mmr, heavy metals, leaky gut, mercury, ethylmercury, thimerosal, preservative, dpt, pharmaceutical companies, big pharma, autism, autistic, asperger, antivax, anti vax, aspie, asd, cdcwhistleblower, cdc whistleblower, sb277, Vaxxed, thalidomide, misoprostol, chlorpyrifos, SB276, pandemic, medical exemptions, influenza, flushot, flu shot, vaccine safe, i vaccinate, immunize, immunization, herd immunity, vaccineinjury, rubella, ivaccinate, pro vax, provax, flu, bigpharma, antivaxx, antivaxxers, vaccineinjured, vaccine injured, vaccine safety, whistleblower, companies, pharma, vaccinate, exemption  b1less, diphtheria, pertussistetanus, Assberger, conscientious exemption, no jab no play, vaccine benefits, vaccine basics, vaccine pros and cons, Consciousexemption, conscious exemption, Vaccinecrisis, Vaccinebenefits, Vaccinebasics, Vaccinefacts, vaccine crisis, Vaccineinjuries, Vaccineworks, valproic acid, Mmrvaccine, Hearus, Vaccinesafe, Vaccinesafety, vaccine facts, rubella virus, vaccine-hesitant, vaccine works, Methylmercury |
| --- | --- |
