# Supplementary material for: Using Machine Learning to Compare Provaccine and Antivaccine Discourse Among the Public on Social Media: Algorithm Development Study
Source: JMIR Public Health Surveill. 2021 Jun 24;7(6):e23105. doi: 10.2196/23105 (PMC8277307; doi:10.2196/23105)
Supplement: Multimedia Appendix 2 [file publichealth_v7i6e23105_app2.docx]

**Multimedia Appendix 2.** Qualitative coding scheme.

| Entman Framework Category | Definition derived from Entman (1993) | Coding Rules for Anti-Vaccine Discussion | Coding Rules for Pro-Vaccine Discussion | Examples of ‘Cold War’ frames derived from Entman (1993) |
| --- | --- | --- | --- | --- |
| Define problems | Determine that a causal agent is creating costs, usually measured by cultural values. This means that the problems cause harm to important cultural values and norms. | Step 1 - Identify if there are any topics related to harmful effects or situations  Step 2 - Identify topics related to cultural value such as medical freedom, safety of children, health, critical thinking, free speech, environmental degradation, greed, etc. that are eroded by the effect or situation  Step 3 - Determine what is the direct cause of the effect/situation. This may or may not be a topic explicitly discussed.  Step 4 – Problem is defined by the cause of the harmful effects/ situation eroding the cultural value | Step 1 - Identify if there are any topics related to harmful effects or situations  Step 2 - Identify a topic related cultural value such as pro-science, health, critical thinking, public health, greed, etc. that are eroded by the effect or situation  Step 3 - Determine what is the direct cause of the effect/situation  Step 4 – Problem is defined by the cause of the harmful effects/ situation eroding the cultural value | “Civil wars are going on around the world”  “America’s influence is being undermined”  “Russia’s place in the world is being undermined” |
| Diagnose causes | Identify the forces creating the problem. All problems have direct or indirect causes that perpetuate the problem. Usually the problem is not stated. | Step 1 – Identify if there are topics related to a person, or organization.  Step 2 – Determine if there is a topic related to a problem/ harmful issue related to vaccines. This may or may not be a topic explicitly discussed  Step 3 – Determine whether there is a link between the person/organization and the problem/ issue.  Step 4 – Cause is diagnosed as a problem/ harmful issue being caused by the person, or organization based on the link between the two  Or  Step 1 – Identify if there are topics related to criticism, mockery, or attacks  Step 2 – Determine that there is NO topic related to the moral judgement of the cause  Step 3 – Cause is diagnosed as critic of anti-vaccinists | Step 1 – Identify if there are topics related to a person, or organization.  Step 2 – Determine if there is a topic related to a problem/ harmful issue that could be solved by vaccines or is not directly implied to be caused by vaccines  Step 3 – Determine whether there is a link between the person/organization and the problem/ issue  Step 4 – Cause is diagnosed as a problem/ harmful issue being caused by the person, or organization based on the link between the two  Or  Step 1 – Identify if there are topics related to criticism, mockery, or attacks  Step 2 – Determine that there is NO topic related to the moral judgement of the cause  Step 3 – Cause is diagnosed as anti-vaccine critics | “Communist rebels are causing civil wars”  “Communists have infiltrated America’s government”  “Capitalists are hurting Russia and communism” |
| Make moral judgements | Evaluate causal agents and their effects. Specifically, there should be a judgment on the problems or causal agents of the problem. The problem itself does not need to be mentioned. | Step 1 – Identify topics that are related to a moral value such as such as medical freedom, safety of children, health, critical thinking, free speech, environmental degradation, greed  Step 2 – Determine whether person/organization is being evaluated based on the moral value  Or  Step 2a - Determine that the moral value is being eroded/harmed  Step 3 – Moral judgement is defined by the moral value being negatively applied to a person/organization OR the moral value being generally eroded. | Step 1 - Identify a topic related moral value such as pro-science, health, critical thinking, public health, greed, etc.  Step 2 – Determine whether person/organization is being evaluated based on the moral value  Or  Determine that the moral value is being eroded/harmed  Step 3 – Moral judgement is defined by the moral value being negatively applied to a person/organization OR the moral value being generally eroded. | “Communists are atheists who are aggressively fighting us”  “Communist infiltrators are traitors and dishonorable”  “Capitalists are greedy and want to take over the world” |
| Suggest remedies | Offer and justify treatments for the problems and predict their likely effects. The solution can be described as being offered by the speaker or as suggestions that others may implement. | Step 1 – Identify topics related to vaccine aligned persons/organizations  Step 2 – Identify topics that can harm vaccine aligned persons/organizations  OR  Step 2a – Identify topics that are related to solutions for stopping vaccine use  Step 3 – remedies include solutions to stop vaccine use OR topics that harm vaccine aligned persons/organizations | Step 1 – Identify topics related to anti - vaccine persons/organizations  Step 2 – Identify topics that can harm anti vaccine persons/organizations  OR  Step 2a – Identify topics that are related to solutions for improving vaccine use  Step 3 – remedies include solutions to improve vaccine use OR topics that harm anti vaccine persons/organizations | “Support U.S intervention in other countries”  “Create a commission to investigate public officials”  “Support Soviet intervention in other countries” |
